# THE EXPERIENCE OF SUBJECTIVE AGE DURING EVERYDAY LIFE

**DOI:** 10.1093/geroni/igac059.646

**Published:** 2022-12-20

**Authors:** Dayna Touron, Matthew Hughes

**Affiliations:** University of North Carolina at Greensboro, Greensboro, North Carolina, United States; University of North Carolina at Greensboro, Greensboro, North Carolina, United States

## Abstract

Empirical work has shown that subjective age is susceptible to momentary fluctuation throughout the day, and in certain contexts like challenging cognitive evaluations. We propose and test a contextual model that describes how momentary experiences impact subjective age, which in turn impacts daily activities and well-being. Using an experience sampling approach, 200 participants were asked to complete 6 surveys per day for a week. Questions asked about the task they were engaged in, including mental, physical and social engagement, challenge, motivation, confidence, and enjoyment. Participants also completed a manipulation of mental challenge and reported their momentary subjective age. Preliminary multilevel models show that certain momentary factors, such as whether participants were enjoying a task or found the task engaging, predict fluctuations in subjective age. We will also discuss moderators, subjective age domains, time of day effects, and the cross-lagged influence of cognitive stressors over time.

